# The discordance between evidence and health policy in the United States: the science of translational research and the critical role of diverse stakeholders

**DOI:** 10.1186/s12961-018-0336-7

**Published:** 2018-08-16

**Authors:** Mohsen Malekinejad, Hacsi Horvath, Harry Snyder, Claire D. Brindis

**Affiliations:** 1 Philip R. Lee Institute for Health Policy Studies, University of California, San Francisco, 3333 California Street, Suite 265, San Francisco, CA 94118 United States of America; 2 Global Health Sciences, University of California, San Francisco, San Francisco, CA United States of America; 3 Department of Epidemiology and Biostatistics, University of California, San Francisco, San Francisco, CA United States of America; 4 Advocacy Leader in Residence, University of California School of Public Health, Berkeley, CA United States of America; 5 Adolescent and Young Adult Health National Resource Center, University of California, San Francisco, San Francisco, CA United States of America

**Keywords:** Evidence-based public health, Health policy, Systematic reviews, Research translation, Legislation, United States

## Abstract

**Background:**

There is often a discordance between health research evidence and public health policies implemented by the United States federal government. In the process of developing health policy, discordance can arise through subjective and objective factors that are unrelated to the value of the evidence itself, and can inhibit the use of research evidence. We explore two common types of discordance through four illustrative examples and then propose a potential means of addressing discordance.

**Discussion:**

In Discordance 1, public health authorities make recommendations for policy action, yet these are not based on high quality, rigorously synthesised research evidence. In Discordance 2, evidence-based public health recommendations are ignored or discounted in developing United States federal government policy. Both types could lead to serious risks of public health and clinical patient harms.

We suggest that, to mitigate risks associated with these discordances, public health practitioners, health policy-makers, health advocates and other key stakeholders should take the opportunity to learn or expand their knowledge regarding current research methods, as well as improve their skills for appropriately considering the strengths and limitations of research evidence. This could help stakeholders to adopt a more nuanced approach to developing health policy. Stakeholders should also have a more insightful contextual awareness of these discordances and understand their potential harms. In Discordance 1, public health organisations and authorities need to acknowledge their own historical roles in making public health recommendations with insufficient evidence for improving health outcomes. In Discordance 2, policy-makers should recognise the larger impact of their decision-making based on minimal or flawed evidence, including the potential for poor health outcomes at population level and the waste of huge sums. In both types of discordance, stakeholders need to consider the impact of their own unconscious biases in championing evidence that may not be valid or conclusive.

**Conclusion:**

Public health policy needs to provide evidence-based solutions to public health problems, but this is not always done. We discuss some of the factors inhibiting evidence-based decision-making in United States federal government public health policy and suggest ways these could be addressed.

## Background

Rigorously synthesised health research evidence should inform clinical practice, guideline development and health policy [1]. Although many gaps and barriers to their use remain, policy-makers, clinicians and patients increasingly rely on synthesised research evidence in healthcare decision-making [2]. This is reflected in the substantial annual increase in systematic review production [3], the emergence of organisations and initiatives promoting evidence-based healthcare decisions [4–8], policies established by funding agencies that mandate use and dissemination of research findings, and generally increased attention by the public and the media to the role of scientific evidence in health policy formulation.

Many lifesaving medical practices across numerous disciplines have been established in recent years, based on evidence derived from rigorous syntheses of the scientific literature. Some noteworthy examples include the use of statins for preventing and treating cardiovascular disease [9], adult male circumcision for preventing HIV in sub-Saharan Africa and other high-burden settings [10, 11], and routine immunisation against myriad communicable diseases [12, 13]. Scientific evidence has also influenced legislation and policy in various arenas. For example, research evidence informed laws to lower blood alcohol limits for motor vehicle drivers [14] and to restrict lead in paint and reduce it in gasoline [15]. Evidence also informed United States Food and Drug Administration regulations regarding the use of industrially produced trans fatty acids from partially hydrogenated oils in food [16, 17]. These laws and regulations have in turn saved countless lives.

However, many competing social values may drive public policy, and better health is only one of them. Health guideline methodologists have recognised that financial or other resource constraints, trade-offs between desirable and undesirable outcomes, feasibility of intervention implementation, variability in stakeholder values, and preferences and uncertainty about the stability of effect estimates are all important factors in determining whether research evidence is translated to health policy [18, 19]. Adding further complexity, any consideration of stakeholder values and preferences must include not only deeply held cultural beliefs, including beliefs about the appropriate role of government, but also other subjective forces such as social stigma. Partisan politics, agendas promoted by interest groups, and donations to policy-makers from industries threatened by new public health regulations, among other factors, may all have an impact on implemented health policy. Above this storm of competing subjective forces, proponents of research evidence may struggle to be heard.

The policy-making process may require judgment calls by a variety of stakeholders. Although their intentions may be good, the judgment of health policy stakeholders can be influenced by several unconscious biases, which may not be obvious or apparent to them. These may include ‘irrational escalation’ (the tendency to justify actions that are already taken or to make irrational decisions based upon past rational decisions) [20], ‘status quo bias’ (the preference to keep things relatively the same) [21], ‘confirmation bias’ (the tendency to search for or interpret knowledge in a way that confirms preconceptions) [22], and ‘observer-expectancy effect’ (the unconscious tendency to manipulate or misinterpret facts in order to support one’s viewpoint about a given expected outcome) [23]. Disentangling how these biases may interrupt the health policy development process or their effects on the final outcome can be very challenging, but every day that they still apply may mean worse outcomes for many, as well as wasted money.

As one of its core functions, government is charged with improving the nation’s health and protecting its citizens from harms caused by natural disasters, environmental threats, and people motivated by self-serving interests. In many circumstances, the government is the only entity with the authority and capacity to protect the public good against competing ideological, economic and other interests [24]. Although there are numerous examples where research evidence was used to formulate health policy and practice to accomplish the common good, there are also many examples where there is a discordance between the available evidence and its application.

We provide four examples to illustrate these discordances and then discuss some reasons (i.e. barriers or interrupting factors) for why research evidence may not end up being reflected in health policy recommendations or in United States federal health policy. We also propose a way to resolve these discordances. We wish to emphasise that this paper is not meant to be a comprehensive review of interventions related to the public health areas discussed here, nor does it provide an exhaustive list of factors and barriers that could interfere with the process of translating the best research evidence into health policy.

## Main text

### Working definitions and conceptual framework

**Fig. 1:**
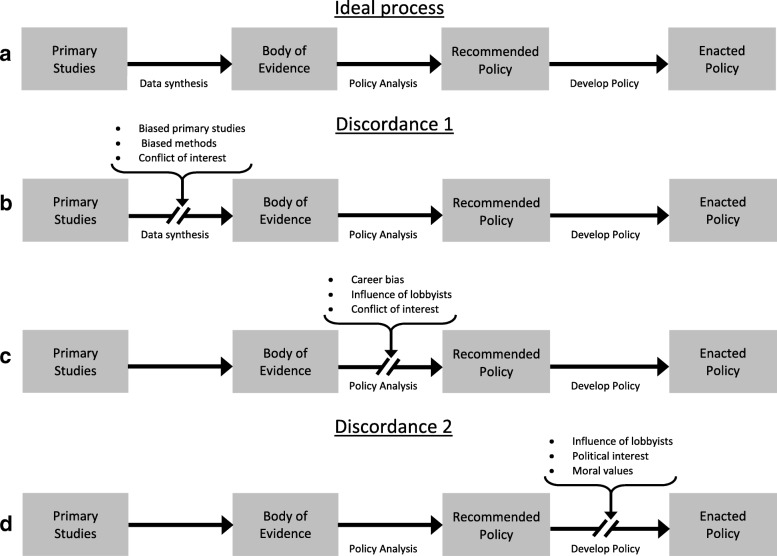
Conceptual framework: evidence-based policy-making process and ‘unwanted’ factors influencing discordance. **a** Ideal process, **b** and **c** Discordance 1, **d** Discordance 2

The following working definitions and conceptual framework (Fig. 1) help to contextualise the role of evidence within the context of competing factors for policy development.

#### Evidence-based public health, epidemiology, systematic reviews

Evidence-based public health has been defined as “*the process of integrating science-based interventions with community preferences to improve the health of populations*” [25]. An important cornerstone of public health is epidemiology, which analyses the causes and determinants of health and illness in populations, the characteristics of public health problems, and the effectiveness of public health interventions. Rigorous epidemiologic methods should be used to synthesise the evidence base for public health policy decisions [1]. High-quality systematic reviews (such as Cochrane reviews) use globally agreed-upon standards and rigorous methods and are widely acknowledged to be the gold standard in approaches to collecting, analysing and critically appraising aggregated research data to inform healthcare and public health decision-making [26].

#### Evidence

There are at least four types of public health evidence that may inform public health policy and practice:

1. *Disease burden:* data about incidence, prevalence and severity of a specific health condition in a specific population and setting. In a rational decision-making process, this type of evidence is used to decide if a health condition’s current or potential burden is sufficiently serious that it merits consideration for health policy development, including establishing regulations that may reduce or eliminate disease risk factors.
2. *Intervention efficacy:* data about how well interventions work to prevent or treat diseases or health conditions. This type of evidence is essential to inform prevention and treatment policies. Ideally, only interventions shown efficacious through rigorous evaluation in a systematic review would be promoted for policy development.
3. *Intervention effectiveness (versus efficacy):* data about circumstances under which an intervention that is proven to be efficacious in the research setting would also work in real-world practice [27]. Examples of such data include intervention delivery modality, data regarding the quality of the programme provided in different intervention settings, and differences in local infrastructure and feasibility of implementation.
4. *Intervention cost and cost-effectiveness*: data about the cost of providing an intervention and its cost-effectiveness, as well as the level of population health improvements gained in relation to its cost. Given that public health resources are traditionally limited, promotion of cost-effective interventions results in greater health benefit for the money invested, compared to other options.

#### Translation of evidence to policy: conceptual framework (Fig. 1)

Several conceptual frameworks and theoretical models have been proposed to portray the lifecycle of research and policy development [28–30], and the discordance that can arise [29, 31].

To break down the steps required in the translation of evidence into policy, we adapted the ‘Policy Process’ framework developed by the Centers for Disease Control and Prevention (CDC) [32] and others [33]. For simplicity, we considered the ideal process (Fig. 1, panel a) of integration of research evidence into health policy as a linear, continuous process, with three major steps:

1. *Data synthesis (from primary studies to the body of evidence):* This is the process through which data from primary studies are collected and analysed via a systematic and transparent process designed to minimise risk of bias and enhance internal validly and precision. Following this process, recommendations can be made in favour of or against an intervention as alternative policy options, with different levels of strength or conditionality regarding the utility of adopting programmes with sufficient evidence.
2. *Policy analysis (from the body of evidence to recommended policy):* This is a process to examine available options using quantitative and qualitative methods to respond to a public health problem. Several frameworks and checklists have been proposed to achieve the goals of public health policy analyses [32, 34, 35]. In addition to technical criteria, such as intervention effectiveness and cost, there may be cultural, feasibility, equity and political criteria to consider in informing policy development.
3. *Policy development (from the recommended policy to the enacted policy).* This is a process for identifying strategies for improving policy adoption and implementation. This process may include development of strategies to engage stakeholders in policy uptake to optimally inform law, regulation or other executive action.

The intention of this framework (Fig. 1) is to facilitate understanding of an evidence-based decision-making model in the context of public health interventions and how knowledge of evidence synthesis (or lack of this knowledge) may influence decision-making. The framework is not intended to be comprehensive nor to replace existing theoretical models. It presupposes the existence of public health problems that should be solved, though there may be a lack of consensus regarding the best course of action.

### The discordance in evidence to policy

If we were to consider an intervention for improving health, there would ideally be concordance between the government’s proposed health policy and guidance (recommendations) given by leading public health agencies about that intervention. Additionally, recommendations for or against the intervention would be made based on high-quality evidence for achieving the desired health outcome and decision-makers would be motivated to incorporate those recommendations in making new or the necessary changes to existing policies and programmes. In the process of evidence to policy translation, however, at least two main scenarios can result in a discordance.

#### Discordance 1

This happens when research evidence does not support the use of an intervention, but public health authorities recommend the intervention nonetheless (Fig. 1, panel b and panel c). For example, it may occur when an intervention to address a public health problem is characterised by authorities as effective, despite low quality evidence, or even when evidence demonstrates that it does not work. This can lead to funding for programmes that are less effective than claimed. At minimum, this results in a waste of financial resources that could have been used for programmes that really do work. Worse, this may result in increased morbidity and associated costs. This type of discordance may arise in the process of data synthesis (Fig. 1, panel b) or policy analysis (Fig. 1, panel c).

- The data synthesis step (Fig. 1, panel b), the process of arriving to a body of evidence from primary studies, can be flawed in several ways. For instance, the results of primary (empirical) studies assessing the effect of interventions may be biased due to weak methodology or investigators’ conflicts of interest, especially when studies are funded by industry [36] or other vested interest groups. In other cases, harms and adverse effects are minimised or completely omitted in scientific literature concerned with a given intervention [37–39]. Study findings, including both benefits and harms of interventions, are often reported in complex and confusing ways. Further, substandard methods are used to gather, synthesise and interpret findings of primary studies. Although it used to be so, it is no longer appropriate simply to assert that systematic review evidence is good evidence [40]. Even when systematic review authors believe they are using rigorous methods to examine intervention effects, their methods may in fact be poor, resulting in untrustworthy findings [3]. For instance, review authors may ‘cherry-pick’ favourable outcomes to create an impression of efficacy, either intentionally, or through poor understanding of methods or unconscious bias.
- The policy analysis step (Fig. 1, panel c), the process for arriving to a policy recommendation based on a body of research evidence and other considerations, can also be adversely influenced. This issue may arise when the review authors themselves are directly or indirectly affected by the implications of the policy analysis. In that case, they may selectively focus on favourable outcomes of certain interventions or even ‘spin’ review evidence to promote an agenda [41, 42]. This selective reporting and outcome-spinning may arise through a sort of altruistic bias associated with unconsciously wanting an intervention to work (i.e. due to confirmation bias or observer-expectancy effect), but there could also be subtle and perhaps borderline conflicts of interest associated with expectations of future funding [43]. Finally, these issues may also arise due to the pressure that public health authorities may face when pressured to come up with solutions to societal problems that politicians and the society want solved. In that case, they may recommend a policy despite a lack of solid and high quality evidence.

Although closely engaged in the policy development process, policy-makers and many other stakeholders may not have sufficient epidemiologic insight to appraise and understand such nuances and several types of bias. All these issues can have a very direct and dynamic bearing on the extent to which research evidence should be believed. Ironically, this may also cause policy-makers to hesitate to rely on evidence.

An ability to understand research evidence is essential to improving health at the population level, but it is only one piece of the puzzle in developing and implementing evidence-based health policy. As discussed below, other interruptive factors and barriers can stop a truly effective intervention from making its way to policy.

#### Discordance 2

This discordance (Fig. 1d) often occurs in two contexts. First, public health authorities may recommend an intervention that is well supported by research evidence, but policy-makers reject it. Despite even high quality and conclusive evidence supporting its efficacy and cost-effectiveness, the intervention is a ‘hot potato’ that policy-makers would rather drop. In some cases, policy-makers may not consider such interventions at all, or only consider them with partial coverage, limited resources or with a delay in implementation. Second, an intervention may hold only inconclusive evidence, yet would still be approved and implemented. This issue could arise when constituents or special interest groups pressure politicians around election time to fix a problem. The politician might then rush into taking actions that would serve short-term political gains, at the expense of giving policy options adequate scrutiny. In both scenarios, the discordance arises through social, cultural and other external considerations (e.g. the influence of special interests) that compete on equal (or even stronger) terms with research evidence [44].

There are other legitimate considerations beyond evidence for an intervention’s efficacy and cost-effectiveness. These may include costs, feasibility of implementation, market dominance and other factors. External considerations, such as sociocultural factors, political influence, interpersonal dynamics and action based on shared misunderstanding, may also come into play as strategic tools for affecting policy adoption. These factors are part of what can be described as an ‘ecosystem’ of health policy development [44]. In an ideal world, this latter group of considerations should not influence the policy itself; health policy should promote an intervention with the highest health impact at the lowest reasonable cost. However, in the real ecosystem of health policy development, even very efficacious interventions with potential for high impact may be shunted aside and then ‘die’ in a legislative committee. Interventions shown to be efficacious in well-controlled study conditions may have very different effects in real-world settings or in populations with different cultural values and norms. Important harms may also arise; the intervention could become ineffective over the long term, or there could be other kinds of undesirable effects. Depending on these variables, it may not always be detrimental that an efficacious intervention is not implemented immediately in all settings. These are all legitimate considerations in a rational decision-making process and in such instances may argue for a longer timeline to fully achieve policy implementation, especially if evidence and data are not available regarding all populations that will be impacted by the policy.

Depending on the specific context, the views of interest groups with fixed ideas about what should be done may prevail over research evidence in the policy-making process. Deeply held ideologies and philosophical positions of deep-pocketed political donors may also be a force. The interests of corporations or even whole industries may be an imposing shadow that looms behind policy decisions [45–47]. General aversion to change, political expediency and unstated conflicts of interest may also serve to exclude research evidence from policy enactment and implementation [48]. Politicians are often unwilling to invest in programmes with long-term returns due to the realities of their short-term election cycles. In the United States federal health policy context, all of these variables are often in play and the result is a fractured and somewhat incoherent health policy landscape.

### Illustrative examples

To contextualise the idea of discordance in the real-life arena of public health policy in the United States, we selected and analysed four diverse public health problems with a relatively high public health burden and/or a significant individual and societal cost. Of the four topics, two reflect discordance type 1 (adolescent pregnancy and adult breast cancer) and two mainly reflect discordance type 2 (childhood obesity and HIV infection in injection drug users). We purposefully selected these four cases because they offer concrete examples for at least one discordance type that was known to us. Given the complexity of the policy-making process, health policy in these areas may be affected by more than one discordance type.

Table 1 provides a summary for the status of the evidence-based interventions addressing these public health issues, current national-level United States policies relevant to these interventions, as well as recommendations made by key United States public health and medical agencies and organisations, examples of related evidence to policy discordance, potential human life and financial losses attributable to the discordances, and interruptive factors and barriers impeding translation of evidence to policy.

**Table 1:** Four illustrative examples of evidence-to-policy discordance in the context of health policy in the Unites States

Health issue	Populations	Evidence	Evidence-based interventions	Discorded policy	Losses due to discordance	Barriers and interruptive factors
DISCORDANCE 1 - CURRENT RECOMMENDATIONS PROMOTE INTERVENTIONS THAT DO NOT WORK						
Example 1: Adolescent pregnancy	Primary: Adolescents Secondary: Children born to adolescents; grandparents of these children	Many systematic reviews of variable quality; United States and international studies Wide range of interventions Mostly inconclusive and conflicting results except for use of contraceptive methods Primary studies of behavioural interventions at high risk of bias, most emphasising self-reported behavioural and ‘knowledge’ outcomes Review conclusions often overstate intervention impact; little evidence of any intervention efficacy in improving biomedical outcomes	High quality systematic reviews only support the promotion of contraceptive use combined with education Insufficient evidence to conclude that any youth sexual risk reduction intervention effectively prevents pregnancy Substantial low-quality evidence exists to suggest that currently recommended interventions have no impact on pregnancy rates	United States government agencies (CDC, OAH) provide millions of dollars in domestic funding for youth sexual risk reduction programmes that have been shown to be ineffective Agencies claim population-level ‘impact’ in reducing teen pregnancy, which is far more likely due to secular trends OAH classify these interventions as ‘evidence based’, but criteria for this determination are very weak and methodologically unsound	Human resource potential (losses to future workforce when adolescents become parents) Personal development potential (education, career and other life goals may be less attainable when adolescents become parents; children born to adolescent parents may live in poverty; grandparents of children born to adolescent parents may take responsibility for raising them) Costs (social services funding of prenatal, postnatal and child healthcare; social services funding for low-income families; government and non-profit funding of programmes that do not work)	Federal government agencies’ use of obsolete and flawed method for evidence synthesis and mischaracterisation of the term ‘evidence based’ to support interventions that have little or no impact on health Unconscious biases induced by, for instance, widespread perception that current interventions are effective in reducing pregnancy, and unwillingness to change that perspective (status quo bias) Inability of policy-makers at all levels to discern that funded interventions are ineffective, swayed by enthusiastic statements of programme implementers and perhaps by their own career bias Unconscious biases, such as widespread perception that current interventions are effective in reducing pregnancy, and unwillingness to change that perspective (status quo bias)
Example 2: Breast cancer	Primary: Adult Women (aged > 40) Secondary: Adult men	Multiple systematic reviews exist including by Cochrane Collaboration [80] and USPSTF [145]. There are at least 8 clinical trials and several observational studies of mixed quality	Mammography for breast cancer screening is considered an evidence-based strategy for women aged 40–70; however, appropriateness of its recommendation is debated	At the federal level, there are programmes providing free mammography to women without access Health insurance companies are required to cover mammography cost for women > 40 years 1–2 times a year Recommendations failed to properly assess the balance between benefit and harms at the population level High quality systematic reviews do show small benefit due to mammography and only in older age groups (50–70) Significant harms associated with the procedure, specially overdiagnosis and overtreatment outweigh benefit	Assuming 30% risk of overdiagnosis with mammography and overtreatment, for every 2000 women participating in screening, over 10 years’ time span, 1 death will be prevented but 10 healthy women will be unnecessarily diagnosed and treated In addition to its cost, mammography has several other side effects including anxiety, pain, risk of further tests and biopsies in false positive cases and risk associated with repeated exposure to x-ray	Role of the advocacy groups that may gain professionally by disseminating false information about the excessive benefit and negligible harms associated with the mammography Lack of knowledge of stakeholders about evidence-based principles in order to help them fully understand nuanced issues around balancing benefits and harms of such intervention
DISCORDANCE 2 - CURRENT POLICY DOES NOT SUPPORT AN EFFECTIVE EVIDENCE-BASED INTERVENTION						
Example 3: Childhood obesity	Primary: Children and adolescents Secondary: General population, as this generation grows older	Many systematic reviews included studies from United States and other developed countries Systematic reviews have summarised evidence by intervention content (diet, physical activity, combination, etc.), setting (school, home, community, combination, etc.), and level (policy and environmental vs. individual) Significant heterogeneity in study measurements, specific content of interventions, and risk of bias Lack of data about certain populations and sex limited external validity of findings	Multiple interventions promoted as evidence based by major public health entities	Lack of a comprehensive and multifaceted national level legislation to address the magnitude of the public health problem	Health and economic consequence of childhood obesity is overwhelming and continues to rise	Childhood obesity is a multifaceted phenomenon, caused by inter-linked cultural, economic, and health and general literacy barriers; however, most interventions promoted as evidence-based tend to apply a bio-medical model and single approach model with possible short-term effect under ideal circumstances with limited applicability outside of tested settings Lobby of food industry since structural level interventions would require major regulatory and restrictive changes of their procedures and practices Complexity of subject matter prohibits non-scientific stakeholders to be able to fully understand what works
Example 4: HIV epidemic among people who inject drugs	Primary: PWID Secondary: sexual partners of HIV-positive individuals and their children	Current: Numerous high quality systematic reviews established the intervention effectiveness in the United States and international settings A systematic review of economic evaluations also supports that SSPs are cost-effective Historical: As early as 1993, several reports and reviews, conducted or commissioned by public health authorities, including the CDC and National Academy of Science, concluded that SSP is safe and reduces HIV transmission [146] Early programmes evaluated in the United States were in Tacoma, WA and Portland, OR [147]	Provision of free sterile needles and other drug injection paraphernalia in various forms such as SSPs This intervention is often combined with other preventive measures such as condoms, peer education and HIV testing and counselling	The evidence-based recommendation has not been fully adopted by the United States federal government as a policy Local and state health departments as well as private donors have funded > 220 programmes in more than 37 states To respond to re-emergence of HIV and hepatitis epidemics among PWID, the Consolidated Appropriations Act of 2015 includes language providing states and local communities, under limited circumstances, the opportunity to use federal funds to support certain components of SSPs, but not to pay for needles and syringes	No comprehensive assessment at the federal level Modelling studies conducted in different cities have shown that thousands of infected cases could have been averted if SSPs were implemented	The argument that the provision of free needles may increase drug use and injection In the absence of RCTs, evidence from other studies was dismissed, and lack of knowledge of the principle of evidence-based medicine may play a role in how some policy-makers understood the evidence HIV epidemic among people who inject drugs coincided with ‘war on drugs’ policy in response to the increased violence associated with crack cocaine use in the 1980s; 47 states classify syringes as drug paraphernalia, making them illegal to buy or own without a prescription Legislators from states with more conservative constituents do not want to be seen as ‘soft’ on drug enforcement or flip-flopping on a matter of moral importance

*CDC* Centers for Disease Control and Prevention, *OAH* Office of Adolescents Health, *PWID* people who inject drugs, *SSP* syringe service programme, *USPSTF* United States Preventive Services Task Force

### Discordance 1: Current recommendations promote interventions that do not work

#### Example 1 – Interventions to prevent pregnancy in adolescents

##### Public health issue

Despite a substantial decrease in adolescent pregnancy over the past two decades, nearly 230,000 babies were born to women aged 15–19 years in 2015 [49]. Teenage pregnancy and childbearing is associated with massive economic and social costs [50, 51]. Teenage pregnancy has serious short- and long-term impacts on the lives of teen mothers, the parents of these girls, as well as their children [50].

##### Evidence for recommended interventions

A wide range of interventions have been designed and implemented to address the teen pregnancy problem worldwide and in the United States [52–54]. Among others, these include educational and behavioural interventions focusing on increasing adolescents’ knowledge about the risk of pregnancy, delaying the age of sexual debut, building contraceptive use skills, promoting consistent use of birth control methods, and providing birth control methods. Among all the existing interventions, findings of high-quality systematic reviews only support the promotion of contraceptive use combined with education as a mean to reduce unintended pregnancy over a medium- to long-term period [55]. Although there are many randomised and observational studies, there is a paucity of evidence supporting population-level impact on pregnancy rates of behavioural sexual risk reduction interventions for adolescents [52]. Lack of evidence is in part due to the use of biased methods, indirect assessed outcomes (e.g. evaluating commonly used proxy outcomes, such as change in knowledge and behaviour, instead of pregnancy itself), inapplicability of content, non-fidelity in replication, and the heterogeneous modalities in which interventions are delivered [52, 54, 56].

##### Policy response

As an example of federal-level policy response to teen pregnancy, we focus on OAH’s Teen Pregnancy Prevention Program. The United States Congress authorised over $101 million each year of its initial programme period (fiscal years 2010–2015) for OAH to make “*competitive contracts and grants to public and private entities to fund medically accurate and age appropriate programs that reduce teen pregnancy*” [57]. After administration and other costs, 75% of the funds were allocated to replicate programmes *“proven*” [sic] to be effective in reducing teen pregnancy, and the other 25% for “*innovative*” programmes [58]. Since 2015, Congress has continued to fund the Teen Pregnancy Prevention Program by allocating $61 million to replicate “*effective*” programmes administrated through 270 cooperative agreements, each ranging from $200,000 to $500,000 per year. For innovative programmes in 2018, $22 million will flow through up to 75 cooperative agreements ranging from $250,000 to $375,000 per year [59–61].

##### Discordance between evidence and recommendation

Despite lack of evidence based on globally accepted standards and practices, behavioural sexual risk reduction interventions are characterised as evidence based and promoted by the OAH in the United States Department of Health and Human Services as a way to prevent teen pregnancy. We argue that, since OAH has used obsolete and arguably flawed methods for synthesising the body of evidence for its pregnancy prevention programmes, it is unacceptable to characterise them as ‘evidence based’ and they should not be recommended for policy. To better understand our rationale, it is important to understand the process and methods that OAH used to evaluate such programmes.

OAH created the Teen Pregnancy Prevention Evidence Review (TPPER) in response to the 2010 Consolidated Appropriations Act [58], mandating that pregnancy prevention programmes must be *“proven effective through rigorous evaluation to reduce teenage pregnancy, behavioral risk factors underlying teenage pregnancy, or other associated risk factors*” [62]. The definition of what the United States Congress understands to be rigorous evaluation is nowhere provided in the congressional appropriations document [55]. It would certainly make sense that they intended for the use of methods that would minimise the risk of any threats to credibility of evidence.

In 2016, OAH released summaries of the results of TPPER evaluation of 25 programmes, and again in 2018. At first glance, the TPPER report appears to be part of an evidence-based decision-making process, but careful examination of the process for generating the summary evaluation leads us to question its rigor. To name a few issues, although some federal agencies (e.g. the Agency for Health Research and Quality (AHRQ)) conduct systematic reviews based on global standard methods, OAH’s programmes are assessed with the simplistic, obsolete methodology developed by What Works Clearinghouse (WWC) at the United States Department of Education more than 15 years ago [63]. Since 2007, WWC’s systematic reviews methodology has been revised by Mathematica Policy Research, a private company, but even with those updates their methods fall short. Among other critiques of WWC’s methodology and Mathematica Policy Research’s errors in assessment [64–66], they received particularly strong criticism in two reports from an organisation called the National Institute for Direct Instruction [67, 68]. The latter critiques suggest that major concerns in WWC systematic reviews, including “*misinterpretation of study findings, inclusion of studies where programs were not fully implemented, exclusion of relevant studies from review, inappropriate inclusion of studies, concerns over WWC policies and procedures, incorrect information about a program developer and/or publisher, and the classification of programs*” [67]. To further expand on the shortcomings of WWC’s methods, we focus on two aspects of their methods and provide examples.

A critical shortcoming aspect of the WWC evaluation method is its low threshold in characterising a programme as evidence based. WWC’s practice is out of alignment with global standards for assessing evidence quality. This is not because WWC’s methods and episteme are uniquely superior. According to WWC, interventions are evidence based if they “*demonstrate evidence of a positive, statistically significant impact on at least one of the following outcomes: sexual activity (initiation; frequency; rates of vaginal, oral and/or anal sex); number of sexual partners; contraceptive use (consistency of use or one-time use, for either condoms or another contraceptive method); STIs; pregnancy*” [69].

In other words, an intervention tested in a study with a finding of one favourable outcome among several neutral or even unfavourable outcomes will be deemed evidence based. For instance, if significantly more 14- to 16-year-old teens at 1-month follow-up report that they have had fewer sexual partners than they reported at baseline, the intervention is deemed evidence based even if 17- to 18-year-old teens reported more partners or if every other outcome of the study was null or negative.

Another shortcoming is really a cluster of concerns with regards to study selection and risk of bias. WWC systematic review methods are idiosyncratic and are not aligned with the rigorous, global standard methods used by the AHRQ and other federal agencies [70]. In determining eligibility of studies for inclusion in WWC systematic reviews, reviewers rate studies according to an algorithm. For example, if a study population was randomised, reviewers next assess whether attrition was high or low. If it was high, they check to see whether study arms were comparable at baseline. If they were not, the study is excluded from the review (deemed “*does not meet WWC standards*”). Had attrition been low but reviewers then discerned unadjusted confounders, the trial would similarly have been excluded [71]. While indeed the evidence from these studies would likely have been of poor quality, reviews conducted according to global standards (e.g. AHRQ reviews) would never exclude poorly conducted studies that in other aspects of population, intervention, comparison, and outcome (PICO) and design met inclusion criteria [70, 72]. Rather, rigorous reviews would assess the risk of bias in each study and report it transparently. It is quite acceptable to exclude studies at high risk of bias from quantitative meta-analyses with studies of similar PICO and design, but WWC excludes these studies entirely from the review without comment [71]. Compounding the problem, WWC reviewers do not formally assess the risk of bias in individual studies. If participants were reported to have been randomised, it does not matter to WWC how well or poorly this was done. Bias associated with lack of blinding and any deficiencies in outcome assessment are also not explicitly considered [73]. There are other serious shortcomings in WWC’s study selection process that are beyond the scope of this paper.

It is not possible to know with certainty the ways in which these problems manifest themselves in OAH’s 2016 summary report [74] or its similar 2018 ‘summary of findings’ [75]. It is also beyond the scope of this paper to explain in detail the differences between OAH criteria and global standards. It may suffice to say that, from the perspective of methods used by AHRQ, Cochrane Collaboration and other leading agencies in the evidence-based public health domain for assessing evidence quality, WWC’s methods seem to be poor [72, 76].

#### Example 2 – Mammography screening for early diagnosis of breast cancer

##### Public health issue

Although mortality attributable to breast cancer has declined substantially since its peak in the 1970s, breast cancer is the most common cancer in women in the United States, regardless of race or ethnicity [77]. In 2014, nearly 230,000 women were diagnosed with breast cancer and approximately 40,000 women died from breast cancer in the United States [78]. The overall risk of breast cancer for women in the United States has not changed in the last decade, though it has increased for some ethnic minorities.

##### Evidence for recommended intervention

Breast cancer screening is generally considered to be part of the standard of care in the battle against the high burden of disease associated with breast cancer among women [79]. The goal of breast cancer screening through mammography is to identify tumours before there are visible signs or symptoms of the disease and to treat cancer early, when chances for cure are higher.

A Cochrane systematic review [80] found seven randomised controlled trials of women aged 39–70 (*n* = 600,000), assigned to receive mammograms or no mammograms. In trials with a low risk of bias, the breast cancer mortality rate was similar in both groups. In trials with a high risk of bias, there were 15% fewer deaths in women receiving mammograms. The reviewers estimated a 30% risk of breast cancer overdiagnosis. Overdiagnosis of breast cancer can lead to unnecessary psychological harm as well as to unnecessary biopsies, mastectomies and deaths. The review’s lead author subsequently published a paper titled “Mammography screening is harmful and should be abandoned” [81]. Subsequent studies showed benefit for screening of women older than 50, however, the best available evidence suggests no benefit for screening average-risk women when they are 40–49 years old.

##### Discordance between evidence and recommendation

Currently in the United States, recommendations for when women should receive mammograms are heterogeneous and vary by organisation or agency issuing the recommendation (Table 2) [82].

**Table 2:** Recommendations about mammography: Women aged 40 to 49 with average risk^a^ [82]

Agency / Recommendation year	Recommendation
United States Preventive Services Task Force (USPSTF) (2016)	“The decision to start screening mammography in women prior to age 50 years should be an individual one. Women who place a higher value on the potential benefit than the potential harms may choose to begin biennial screening between the ages of 40 and 49 years”
American Cancer Society (2015)	“Women aged 40 to 44 years should have the choice to start annual breast cancer screening with mammograms if they wish to do so. The risks of screening as well as the potential benefits should be considered. Women aged 45 to 49 years should get mammograms every year”
American College of Obstetricians and Gynecologists (2011)	“Screening with mammography and clinical breast exams annually”
International Agency for Research on Cancer (2015)	“Insufficient evidence to recommend for or against screening”

^a^Reproduced from the Table of Breast Cancer Screening Guidelines for Women generated by the US Centers for Disease Control and Prevention

Screening for a disease at the population level may be appropriate when, among other conditions, the disease burden is very high, screening tests are reasonably accurate (in terms of both sensitivity and specificity, analysed together), the risk of adverse events is low and costs are low; it may not be appropriate in other contexts. Breast cancer screening is associated with several common and important adverse effects, as follows: (1) a 5–50% risk over a 20-year period of receiving false positive results, leading to more tests that are costly, time-consuming and may cause anxiety [83, 84]; (2) overdiagnosis and overtreatment, namely finding and treating a tumour that would not have gotten worse had it not been detected (overtreatment can have severe side effects, including invasive unnecessary biopsy and mastectomy, radiation therapy, anxiety and even death); (3) procedures; and (4) potential risk of developing new cancers associated with repeated exposure to x-rays [82].

A systematic review of 59 reviews published between 2000 to 2015 about benefits and harms of the mammography concluded that “*the specific expertise and competing interests of the authors influenced the conclusions of systematic reviews*” [36]. The authors reported that, compared to those conducted by non-clinicians, systematic reviews conducted by clinicians significantly reported conclusions favouring mammography.

The American Breast Cancer Foundation seemingly ignores these risks and harms, instead making such recommendations as the following: “*Women should begin scheduling their annual mammograms at the age of 40*” and “*Mammography can help to reduce the number of deaths from breast cancer among women ages 40–70*”, referencing to the CDC surveillance SEER in 2002–2008 [85]. Other breast cancer advocacy organisations also downplay or fail to accurately communicate the risks associated with mammography.

The two examples presented above for Discordance 1 serve to illustrate how lack of knowledge, unconscious biases, vested interests and other factors can have an impact on the validity and reliability of synthesised research evidence underpinning public health recommendations. With that in mind, we now turn our attention to two additional illustrative examples for Discordance 2, which show the types of pressures that often compete with research evidence in health policy development.

#### Example 3 – Interventions to prevent obesity in children

##### Public health issue

Approximately 30% of children and adolescents in the United States are clinically obese (body mass index ≥ 30%) or clinically overweight (body mass index ≥ 25% to < 30%). Children with obesity are at higher risk of developing asthma, type 2 diabetes, bone and joint problems, sleep apnoea, and of becoming obese as adults [86]. This increased rate of obesity has been attributed to increased consumption of sugar-sweetened beverages (SSB), increased use of junk food and other unhealthy foods (high in fat, salt and sugar), decreased physical activity and other factors [87].

##### Evidence for recommended interventions

Several recent systematic reviews provide compelling evidence about a growing number of interventions to prevent childhood obesity. Prevention interventions are diverse in terms of programming (diet, physical activity or both in combination) and setting (home, school, community, child care, primary care or combinations of these settings). A 2013 systematic review concluded that “*physical activity interventions in a school-based setting with a family component or diet and physical activity interventions in a school-based setting with home and community components have the most evidence for effectiveness*” [88]. Another systematic review conducted by the Robert Wood Johnson Foundation identified 12 discrete, physical activity strategies and 13 nutritional interventions that could be implemented through health policy or in environmental designs of schools and community. These include environmental modifications in schools, neighbourhoods and communities that could potentially encourage greater physical activity, as well as prompts for children to begin physical activity [89, 90]. However, to tackle childhood obesity at the population level, a multi-pronged, comprehensive and cohesive set of policies is necessary to address the root causes of the epidemic. Table 3 shows numerous structural [91] approaches that could potentially be deployed in a coordinated fashion to reduce childhood obesity, ranging from interventions through changes in laws and regulations, those operating by means of environmental changes and other designed to influence social norms.

**Table 3:** Potential interventions to reduce childhood obesity [91]

Level	Intervention
Laws and regulation	• Pricing and taxing in favour of healthy food versus junk food • Zoning regulation to control density of fast-food and other unhealthy food outlets in communities • Regulating food advertising to children • Required provision of caloric and nutritional content to consumers in restaurants and fast-food outlets • Required counter advertising to show true impact of unhealthy food
Environmental	• Increasing exposure and availability of healthy food in community and school settings • Environmental modifications to schools, streets and communities
Social norms	• Decision prompts to discourage sedentary behaviour • Making physical activities “*easier, safer, and more attractive*” [148]

Previous policy recommendations, such as after-school physical activity programmes, taxation on SSB, and bans on fast-food TV advertising targeting children have been studied via microsimulation analysis. Of these, the single most effective strategy was increased taxation on SSB [92].

##### Policy response

Childhood obesity has received substantial public health attention in the past decade. By 2013, 30 states in the United States had enacted legislation to create or expand obesity-prevention efforts in children. Currently, the most important federal law with indirect implications for childhood obesity is the Healthy, Hunger-Free Kids Act (2010). This legislation includes six large nationwide programmes [93], but the focus of the legislation is good nutrition for low-income mothers and children, not obesity prevention.

##### Discordance between evidence and recommendation

Children are directly targeted by the fast-food industry, which uses advertising and marketing strategies designed to capture children’s attention. Strategies include the use of cartoon characters, movie stars and sports figures in marketing as well as offering complementary toys with a child’s meal and special play areas at restaurants. Regulating advertisements directly targeting children could be a promising approach to preventing childhood obesity. There is compelling evidence that the food industry creates obesogenic environments to influence children’s preferences for and consumption of foods that contribute to obesity [94]. In 2005, the Institute of Medicine recommended that food-industry advertising that targets children should be eliminated, but little progress has been made since then [13]. Despite a large body of evidence supporting the effectiveness of several interventions, we did not identify any comprehensive legislation that addresses the magnitude of the United States childhood obesity epidemic. A 2015 Congressional bill developed by the United States House of Representatives specifically to stop obesity in schools, the Stop Obesity in Schools Act of 2015, mandates the Department of Health and Human services “*to develop a national strategy to reduce childhood obesity that: (1) provides for the reduction of childhood obesity rates by 10% by the year 2020; (2) addresses short-term and long-term solutions; (3) identifies how the federal government can work effectively with entities to implement the strategy; and (4) includes measures to identify and overcome obstacles*” [95]. The last available record indicates that the bill was referred to the Subcommittee on Early Childhood, Elementary, and Secondary Education on March 23, 2016. As of this writing, no further action has been taken.

#### Example 4 – Interventions to prevent HIV in injection drug users

##### Public health issue

The HIV epidemic remains a major public health challenge in the United States and globally [96]. People who inject drugs (PWID) may share drug paraphernalia or engage in high-risk sexual behaviour, putting them at increased risk of blood-borne infections such as HIV and hepatitis C virus (HCV) [97, 98]. In 2013, over 103,000 men and nearly 70,000 women in the United States were living with HIV, with their acquisition of the virus attributed to injecting drug use [96]. Although in the past few years the rate of HIV diagnoses in PWID has declined by nearly half [99], there has been an increase in the numbers of new heroin injectors each year, notably in the Appalachian region [100], who are now at risk for blood-borne infections through high-risk practices [101], and who also have poor access to HIV and HCV prevention and treatment programmes [102].

##### Evidence for recommended intervention

Along with methadone maintenance treatment, the use of syringe service programmes (SSPs) is an effective strategy to prevent the spread of blood-borne infections in PWID [103]. Provision of clean needles and syringes prevents PWID from sharing these and decreases the risk of HIV and HCV as well as other adverse outcomes [104]. Even 20 years ago, many developed and developing countries worldwide had already implemented SSPs in large scale, but the United States has not done so [105]. There is a large body of evidence generated from empirical and modelling studies supporting the effectiveness of SSPs [104, 106, 107] and cost-effectiveness in the United States [108, 109].

##### Policy response

In 1988, the Department of Health and Human Services forbade the use of any federal funds to support the SSP until it was proven to be safe and effective [110]. Since as early as 1995, CDC and most other public health organisations involved in responding to the HIV epidemic have recommended the provision of free needles [111]. Some states changed their laws permitting syringe exchange programmes in 1990, many years after other countries [112], but until 2015, the United States federal government maintained its total ban on SSP funding. With the recent emergence of an epidemic of new heroin injectors [100], the federal government changed its position to permit funding of SSPs in 2016. However, this funding cannot be used to purchase syringes or other drug paraphernalia [113].

##### Discordance between policy recommendation and enacted policy

Socially conservative members of the United States Congress have disregarded evidence supporting the provision of SSP for PWID for over two decades. In the absence of randomised controlled trials, opponents of the intervention argue that there is no proof that it is effective and safe. They often referred to early studies in Canada (Montreal, Quebec and Vancouver, British Columbia), showing no difference in HIV incidence between needle exchange groups and control groups, and suggested that provision of free needles may increase drug use and injection [114]. However, systematic reviews of numerous subsequent domestic and international empirical studies, as well as modelling and cost-effectiveness analyses, have shown these concerns to be without merit [104, 106–109].

Some time lag between the production of science and its translation into policy and programmes is reasonable and should even be encouraged. In the context of public health programmes such as SSPs, potential harms of the intervention should be given just as much attention as the benefits. However, more than 20 years of research evidence shows that SSPs have minimal harms, while providing significant health and economic benefits.

External considerations beyond the realm of scientific evidence have driven SSP policy decision-making [114, 115]. The historical context helps to explain why this is the case. The early HIV epidemic among PWID coincided with the emergence of the United States ‘War on Drugs’ policy in the 1980s [116]. The War on Drugs was a punitive approach to law enforcement and justice that saw all illicit drug users as criminals, rather than as patients in need of care [117]. It is still very much a part of United States public policy, notwithstanding recent decriminalisation of marijuana laws in some states. Many conservative policy-makers (and their constituents) see PWID as criminal addicts, mere ‘junkies’ who engage in lifestyle choices that they can and should control [118]. This attitude is one reason that many legislators have ignored countervailing research evidence about the efficacy of SSPs in reducing the risk of HIV infection.

##### Key interruptive factors and barriers in the health policy process, in the context of four discordance examples

For simplicity and illustrative purposes, we designate each health policy example to demonstrate one of the discordance types and related interruptive factors or barriers. However, even among these examples, there could still be other interruptive factors that also interfere with the translation of evidence to the policy process, causing Discordance 1 and/or or Discordance 2. There are many other potential factors that we cannot explore here, however, we do suggest that perhaps the three most prominent barriers and interruptive factors in the context of our examples are the lack of knowledge about the principles of evidence-based medicine, unconscious biases, and vested interests and beliefs.

Knowledge of the principles of evidence-based health policy is a cornerstone of evidence-to-policy translation. We have already discussed how the lack of such knowledge among stakeholders may lead to Discordance 1, wherein an intervention may be characterised as evidence based when, in fact, it is not, and even an intervention that results in net harms may be promoted. This may also lead to or facilitate Discordance 2 situations. For instance, we can safely assume that most legislative staffers involved in the process of policy development have not been trained in the principles of evidence-based medicine concepts to the extent that that they could properly understand, appraise and interpret health research evidence. Given the complexity of the evidence base in childhood obesity (second example), even well-trained persons might have difficulty in appreciating nuances of evidence quality. With the fourth example, one needs a relatively strong knowledge base of epidemiologic bias to proceed with good discernment in decision-making.

Unconscious bias may inform our decisions in different ways to those we have already discussed to this point, and can lead to both Discordance 1 and Discordance 2. For example, it is plausible that some stakeholders involved at different stages of policy development might generally prefer to keep things the way they are (status quo bias). Perhaps unaware of global standards, those reviewing the evidence for OAH may prefer to continue using the same biased but familiar methodology. Advocacy groups promoting access to mammography for women may prefer the existing pro-mammography guidelines, given that women’s struggles to increase health coverage where especially relevant to women. Policy-makers may feel the issue of childhood obesity is too complex to tackle; they may feel less pressure to scale up SSPs, compared to current, urgent discussion about the prescription opioid epidemic. They also may not be willing to change their previous public positions, as constituents may see this as flip-flopping on a matter of moral importance. Other types of unconscious biases (e.g. observer-expectancy bias, confirmation bias) can potentially be discerned in any number of health policy examples beyond those that we discuss.

Finally, perhaps the most widely acknowledged interruptive factor in evidence-based policy-making is the influence of vested interests and beliefs. This can creep into every aspect of health policy development. Although decision-makers are typically required to disclose all potential financial conflicts of interest or to affirm that they have none, it is still fair to inquire about potential conflicts of interest, perhaps not strictly financial in nature, when cherry-picked data are used to suggest that a given behavioural intervention works, while evidence for alternative approaches is ignored. What would happen to a professional organisation if it intensively ran campaigns to enroll women over age 40 for mammograms because they purportedly save lives, if that same organisation later had to admit to these women that it had made a mistake? This professional call to action may only have saved lives in a small minority of women while it failed to inform women of potential harms such as the increased incidence of false positive results that likely led to overtreatment. In another controversial area, how could politicians whose constituents perceive injection drug use to be morally degenerate behaviour develop the political willpower to scale-up SSP in the United States? Careful consideration of the types of evidence and data that exist to support or refute existing arguments may be helpful to bring to the public for their education.

## Discussion

We identified two types of discordances between evidence and policy implementation and illustrated their impact with examples. In addition to being wasteful of scarce financial resources, both Discordance 1 (the discordance between research evidence and policy recommendations) and Discordance 2 (discordance between policy recommendations and actual policy) can lead to large negative impacts on human health and wellbeing.

### Harms associated with the discordances

The first harm reflects missed opportunities to save lives by implementing policies driven by our core values and supported high-quality evidence. When there is discordance between policy recommendations and enacted policy, we fail to implement interventions that have been shown to work. Amid conflicts about best approaches and the pressure of other agendas competing against research evidence, important public health problems as prominent as the HIV/AIDS epidemic may be neglected [119]. Policies that go completely against research evidence, such as the war on drugs, may also be enacted [120, 121], which can result in outright societal and individual harms [122, 123].

The second type of harm is reflected in opportunity costs. Public funding is usually limited and there are always competing (e.g. obesity, diabetes) and emerging (e.g. Zika, opioid addiction) public health problems and crises. Thus, for instance, the $1.3 billion public funds spent annually for mammograms in younger (age 40–49) women with only an average risk for breast cancer is funding that might have been better spent on interventions that actually saved women’s lives. Far from competing over scant resources and debating which dreaded disease is more important, the bottom line is the urgent need to make policy decisions that consider policy options based on the best evidence available. Continuing failure to do so supports political narratives that government is the problem, as it is perceived, by some, not to make wise investments, while others perceive that government should not play a role in this type of societal investment.

Physical or psychological harms are a third type of problem. Many medical procedures and public health interventions are accompanied by unintended negative consequences. Even procedures or interventions believed generally to be harmless may have an important negative impact, because the impact of these harms may be downplayed or ignored by investigators [39, 124, 125]. In many cases, harms are not thoroughly assessed and not initially detected in research studies that test an intervention under controlled conditions, but these harms may emerge when delivered at large scale under real-world conditions. For example, licensed drugs believed to be highly effective as treatments, with minimal harms, may later be recalled due to serious side effects [126].

Finally, the discordance between research evidence and policy recommendations can work to discredit scientific efforts and the research community as they are perceived by the general public and policy-makers. The scientific community has always had to fight against ideologies and anti-scientific dogma, especially in the context of public policy. In addition, special-interest groups may actively deploy tactics to discredit scientific findings that are counter to vested interests or beliefs [127–129]. The integrity of the scientific community and of science itself plays an important role in keeping public opinion aligned with scientific fact instead of with hype, spin, conspiracy theory, ideology and other so-called ‘alternative facts’. If those involved in the production, analysis and interpretation of evidence fail to maintain an unbiased view, or even have their own vested interests in the forefront, science and scientific evidence will suffer in the public eye. Further, if scientists overstate their findings, fail to fully interpret results, or fail to convey that science is the result of centuries of constant learning that has evolved over time, the argument for relying on evidence is diminished.

### Role of key health policy stakeholders

There are many stakeholders in health policy development. Besides policy-makers, they may include healthcare providers, insurance companies, government and regulatory agencies, professional disease-specific associations (e.g. American Heart Association), community and grassroots organisations, individual patients and their families, national-level foundations (e.g. Robert Wood Johnson Foundation), lobbyists, product manufacturers, patent holders, the media, and other interest groups. If scientific evidence is used appropriately in the process of policy development, it can potentially have a large impact on population health. This may be seen reflected in laws and regulations that set standards for the quality and safety of our food, the water we drink and the air we breathe, as well as in the quality and quantity of healthcare services we receive.

Within this milieu, health advocacy groups and those engaged in the development and translation of scientific evidence for policy recommendations have a critical role to play in advancing an agenda for improving health. Health advocates are expected to make positive changes by improving access to quality care and preserving patient’s rights at the structural level (i.e. in policies, laws and regulations). Their role is even more crucial for underserved and marginalised communities (e.g. working poor, undocumented immigrants, substance users, homeless populations) whose voices may not be heard in the absence of active advocacy, as well as for topics that may be stigmatised or be controversial, such as SSPs and abortion. However, as with other types of stakeholders, unconscious biases (e.g. confirmation bias) or natural human altruism may also play a role – we want an intervention to work, even when the evidence does not show this conclusively.

Despite this important role, health advocacy has only recently been recognised as a distinct discipline within the domain of public health [130]. However, without a background in epidemiology, advocates may not have a nuanced appreciation of how synthesised research evidence may be used as a means to increase their effectiveness in promoting health.

Some policy-makers and other stakeholders may have training in epidemiology yet lack the sophistication to understand the nature of bias in research or to have an adequate understanding of how to interpret systematic review findings. This is important, since even systematic reviews may promote certain agendas [3, 131] or reflect the biases of programme implementers [41, 42]. When they are told that an intervention is evidence-based, policy-makers may not closely scrutinise the underlying research. Even when these stakeholders are fully versed in all public health dimensions of a given policy decision, their grasp of whether research evidence is strong may be distorted due to prior ideological commitments, organisational influence and even self-interest.

### Future implications

As a first overarching step, the research community should work with other stakeholders involved in health policy development to build a climate in which research evidence is highly valued but also tested rigorously. Often, efforts are made to discredit science or regulatory and public health safety agencies are pressured to downplay scientific evidence. Funding agencies, academic institutions, researchers, scientific journals and others directly engaged in producing and disseminating evidence can play a critical role in overcoming some of these challenges. Among other potential approaches, they could raise the bar in favour of quality versus quantity of research studies, improve the quality of the peer-review process on both ends of the evidence production pipeline (i.e. both research proposals and eventual manuscripts), and train more experts in the field of evidence-based public health. For instance, in the example of behavioural interventions to prevent teen pregnancy, there are too many studies assessing small-size programmes with short-term follow-ups that only measured changes in knowledge and self-reported behaviours (as opposed to an actual pregnancy outcome) of participants. Further, emphasis should be placed on the importance of change in actual pregnancy outcomes; the observed effect on self-reported sexual behaviour outcomes is unreliable and provides indirect evidence. Training of experts in the field of knowledge transfer may also narrow the gap in evidence to policy translation. Knowledge transfer is an emerging field aimed to optimise the transfer of the latest research evidence and stakeholder perspectives, with the goal of improving health outcomes [2]. Health researchers may immunise themselves by practicing high standards of scientific integrity, actively engaging in health advocacy and interactions with diverse stakeholders and policy-makers to communicate their findings, and understanding the culture and values surrounding the problem and policy in question in order to more effectively communicate the scientific evidence to a broader audience outside of the scientific community.

Further, the scientific community must do a better job in educating the public about the fact that science is an evolving process and is thus subject to change. Today’s best evidence may be tomorrow’s old news. Problem solving and policy development require an iterative process. Often, it requires several cycles of planned data gathering and evaluation in order to achieve increasingly better outcomes. Policy-makers often want to declare, ‘problem solved’. Scientists should be willing to say, ‘Let evidence indicate if we are on the right path, and let us be ready to learn and continue to improve upon our outcomes’, and continue to test the applicability of the evidence to the field.

It can also be difficult to identify appropriate and applicable research evidence for policy development. For instance, in respect to childhood obesity, there is a mismatch between the complex and multi-factorial nature of the problem (i.e. caused by interlinked cultural, economic, health, literacy, and other barriers among underserved populations in real-life settings) and the existing evidence (i.e. based on single biomedical interventions in a selective population in a controlled condition).

Another overarching issue is the explosion of research production, making it even harder to differentiate high-quality and relevant evidence from the rest for decision-making. Thousands of new medical and public health articles are indexed in PubMed, alone, each week. Workers in the field of knowledge transfer use synthesised research findings from high-quality systematic reviews to deliver best evidence to policy-makers and other key stakeholders. This evidence may be packaged in the form of ‘evidence briefs’ or other approaches [44, 132], and customised in other ways to meet the needs of specific stakeholder groups.

To minimise harms associated with the discordance between evidence and policy, we propose that those engaged in the policy-making process, in particular those who translate evidence for policy recommendations and who are health advocates, should learn to appraise the evidence informing their policy agenda (e.g. via inclusion of this type of analytic framework and science in their health policy and advocacy curriculum), or work closely with those who do have those skills and can represent the public interest in the policy arena being debated. Health advocates often simplify research findings in order to communicate the essence of the analysis. Trusted advocates should work with the researchers involved to ensure that the statement of the findings is indeed accurate.

We suggest that a minimum grounding of stakeholders in core principles of evidence-based public health, as well as in the science of communicating about research findings, may build a bridge between the latest scientific evidence and public health policy. This may be especially useful when the evidence is not clear-cut, as with pregnancy prevention interventions or mammography for breast cancer. A more nuanced understanding of the degree to which one may believe research findings, including consideration of epidemiologic biases, applicability to the policy question and the role of industry or interest group funding, could help to make United States health policy more evidence-based and improve health at the population level.

In the end, enhanced training may only improve a subsection of the problem that is attributable to lack of adequate knowledge in evidence-based public health. We also need to recognise the importance of building competency in other areas, as outlined below.

We propose more attention be given to improve the communication capacity of policy-makers in the manner that evidence is framed for different audiences, particularly segments that may discount the validity of evidence that may not support findings contrary to these groups’ belief systems or sense of morality, such as the SSPs for HIV prevention. It is particularly crucial that these groups be engaged, especially if they question the importance of making societal investments in particular issues, for example, segments who discount the role of government in developing and implementing programmes.

Competencies should be built to identify common ground across different audience segments, framing results as a means of responding to concerns across different groups concerned with particular societal outcomes, or those who may be concerned with costs related to certain outcomes. They also have to face their own ‘unconscious bias’ in championing evidence that may not actually be valid. Regarding the example of mammography for breast cancer, for instance, different healthcare provider and patient advocacy groups perceive and act upon the same evidence to push their respective agendas forward in different ways.

### Analysis limitations

There are some limitations to our analysis. It is not a comprehensive analysis of all possibilities for discordance; indeed, we acknowledge that the types of discordance we examine here are only two very prominent ones. We selected our examples purposively, and there may have been better exemplars. A couple of other possibilities include screening for depression and primary care check-ups. Depression screening often leads to overdiagnosis and, in most cases, to diagnosed patients initiating antidepressant regimens [133–135], despite many significant known harms from antidepressant medications [38, 136] and side effects that include an increased risk of suicide and violence [39]. Primary care check-ups have been shown to have limited impact on reducing the risk of morbidity or mortality [137] in patients without other serious health risks. However, given the face validity of such interventions and their popularity among patients and advocacy groups, policy-makers may feel compelled to endorse polices that ignore this evidence.

There are likely many other factors causing or mediating Discordance 1 and 2 in the context of our examples. To illustrate each example, we focused our analysis on a single Discordance that was most visible to us, and apparent in the literature. There may or may not also be the complementary Discordance in those contexts. For example, in the example of adolescent pregnancy prevention, we merely reflect on Discordance 1 (e.g. use of inferior data synthesis methods and cherry-picking of favourable findings to characterise behavioural interventions as evidence based). However, it is also likely that this Discordance exists because of the macro-level political and cultural climate in which the alternative approaches (i.e. promotion of contraceptives and abortion services that are truly evidence based) are morally unacceptable for some policy-makers and their constituents, and thus are removed or downplayed in the policy agenda (Discordance 2).

Further, in the context of provided examples, there could be discordance between evidence and policy in respect to other interventions that stakeholders inappropriately promote or demote. For example, in addition to behavioural interventions, OAH has also been promoting certain abstinence-based programmes to prevent teen pregnancy [138, 139], despite lack of evidence of efficacy [52, 55].

In order to properly tease out the actual factors causing or mediating Discordance 1 and Discordance 2 in the context of our examples, we would need to conduct a survey of all stakeholders involved (e.g. OAH, American Breast Cancer Foundation, policy-makers, etc.). Such a study would allow us to collect and analyse primary data about stakeholder knowledge of evidence-based medicine, their deeply held values and beliefs in the context of the subject area, subtle or indirect conflicts of interest that may have bearing on decisions, and other issues that may interfere with the process of rigorously translating evidence to policy. Without such data, we may not be able to get an accurate and comprehensive picture of the real issues from the existing published literature and our analysis may gravitate towards speculation.

## Conclusion

Public health policy should provide evidence-based solutions to public health problems. National and local policy-makers may face barriers in the use of research evidence when allocating resources [140]. Their priorities may be based on obsolete or incomplete evidence or factors other than research evidence.

In both types of discordance that we discuss here, there is a risk of increased population morbidity and/or mortality. It may not be feasible or, indeed, possible to change many context-specific barriers in the use of health research evidence in public health policy and programming. However, we can still mitigate the risks to population health if all stakeholders involved in guiding, developing and implementing public health policy have at least foundational skills in assessing evidence quality, as well as in communicating about it in nuanced ways. This could help to increase the use of the best evidence, which could result in better population health. Even where research evidence and evidence-based recommendations are used only selectively, health policy decisions will at least be evidence informed, if not always evidence based. Given the intractable or systemic nature of many discordances, even this partial uptake could help to reduce harm and improve health at the population level.

Research evidence is often shunted aside in health policy development, but it need not be this way. If research evidence were developed by scientists who followed global best practices in conducting and reporting their research [18, 26, 72, 141–144], if health recommendations were made by public health authorities who truly understood the evidentiary strengths and limitations of the research under examination, and if policy-makers could, in turn, do the same, public health and clinical care in the United States would have an increased likelihood of improving significantly.

## Abbreviations

AHRQ: Agency for Health Research and Quality
CDC: Centers for Disease Control and Prevention
HCV: Hepatitis C virus
OAH: Office of Adolescent Health
PWID: People who inject drugs
SSB: Sugar-sweetened beverages
SSP: Syringe service programmes
TPPER: Teen Pregnancy Prevention Evidence Review
WWC: What Works Clearinghouse
